# Real-World Evidence on Use of Ceftazidime-Avibactam in the Management of Gram-Negative Infections: A Retrospective Analysis

**DOI:** 10.7759/cureus.70234

**Published:** 2024-09-26

**Authors:** Subhash Todi, Prachee Sathe, Ramasubramanian V, Subramanian Swaminathan, Deepak Talwar, Parikshit Prayag, Polati Vishnu Rao, Kirti Sabnis, Shweta Kamat, Akshata Mane, Harish Thanusubramanian

**Affiliations:** 1 Department of Critical Care Medicine, Advanced Medical Research Institute (AMRI) Hospital Dhakuria, Kolkata, IND; 2 Department of Critical Care Medicine, Ruby Hall Clinic, Pune, IND; 3 Department of Infectious Disease, Apollo Hospitals, Chennai, IND; 4 Infectious Disease, Gleneagles Global Hospitals, Chennai, Chennai, IND; 5 Department of Medicine, Metro Hospital Noida, Noida, IND; 6 Transplant Infectious Diseases, Deenanath Mangeshkar Hospital, Pune, IND; 7 Infectious Diseases, Apollo Hospitals, Hyderabad, IND; 8 Infectious Diseases Tropical Medicine, Fortis Hospital, Mumbai, IND; 9 Medical Affairs, Pfizer India, Mumbai, IND

**Keywords:** antimicrobial therapy, ceftazidime-avibactam, clinical improvement, gram-negative organisms, microbiological success

## Abstract

There is limited real-world data from India examining the treatment characteristics, safety, and efficacy of ceftazidime-avibactam against Gram-negative organisms especially multidrug-resistant pathogens including carbapenem-resistant *Enterobacterales* and carbapenem-resistant *Pseudomonas*. In this retrospective study, the real-world treatment patterns, effectiveness, and safety of ceftazidime-avibactam in treating Gram-negative infections were assessed. Data was extracted from electronic health records of adult patients admitted to the hospital with documented Gram-negative infection who had received treatment for at least 48 hours with ceftazidime-avibactam as a part of routine clinical management. Among the 189 patients, on Day 3, clinical symptom improvement was recorded in 79.6% of patients who received ceftazidime-avibactam within 72 hours of hospital admission. Clinical success was achieved in 79.5% and 76.3% of assessed patients on Day 7 and Day 14/end-of-treatment (EOT), respectively. Microbiological success was reported in 76% of patients on Day 7 and in 60.3% of patients on Day 14 or EOT. The mean treatment duration of ceftazidime-avibactam therapy was 6.92 (± 4.1) days. No new safety concerns were identified. In conclusion, this study provides real-world evidence on treatment patterns and clinical outcomes associated with ceftazidime-avibactam in India, complementing the previously reported literature. The results suggest ceftazidime-avibactam is an effective and tolerable option for the management of multidrug-resistant (MDR) Gram-negative infections in critically ill patients.

## Introduction

Infections caused by antimicrobial-resistant organisms, particularly multidrug-resistant (MDR) pathogens, impose a major challenge on healthcare systems across the world. An increase in antimicrobial resistance (AMR) leads to increased mortality, hospitalization costs, and length of hospitalization [1]. It is estimated that by the year 2050, Asia will have 4.7 million deaths that could be directly attributed to AMR. AMR is increasing in India with up to 12-59 % of *Escherichia coli* being extended beta-lactamase (ESBL) producers and up to 30% being carbapenemase producers (CP)[2]. Infections caused by MDR Gram-negative bacteria contribute to a high hospitalization rate in low- and middle-income countries [3]. In India infections caused by Gram-negative bacteria have been observed to be associated with higher mortality rates as compared to infections caused by Gram-positive bacteria (17.7% vs. 10.8%)[1].

Among the infections caused by Gram-negative bacteria, the most common drug-resistant organisms are *Enterobacterales* (*E. coli*, *Klebsiella pneumoniae*), *Pseudomonas aeruginosa*, and *Acinetobacter baumannii* [3]. Carbapenem resistance (CR) rates significantly varied with different organisms (30%, 40%, 25%, and 70% for *E. coli*, *K. pneumoniae*, *P. aeruginosa*, and *A. baumannii*, respectively) [2,3]. The epidemiology of resistance mechanisms also differs from country to country. *K. pneumoniae* carbapenemase (KPC) is the most frequently isolated resistance mechanism in the United States and is widespread in South and Central America, the Middle East, and China [4]. On the other hand, for *Enterobacterales*, in India, OXA-48, OXA-48 coproducers, and NDM + OXA-48 are more frequently isolated, while KPC or VIM are uncommon [5,6].

Ceftazidime-avibactam is a combination of the third-generation cephalosporin ceftazidime and avibactam, a non-β-lactam β-lactamase inhibitor. Avibactam has a broad spectrum of activity, inhibiting Ambler class A (e.g., TEM-1, CTX-M-15, KPC-2, KPC-3), class C (e.g., AmpC) and certain class D β-lactamases (e.g., OXA-10, OXA-48) but is not active against class B enzymes (metallo-β-lactamases)[7]. Ceftazidime-avibactam is approved in India for the treatment of complicated intra-abdominal infections (cIAI), complicated urinary tract infections (cUTI), including pyelonephritis, hospital-acquired pneumonia (HAP) including ventilator-associated pneumonia (VAP), caused by susceptible Gram-negative bacteria, and bacteremia secondary to above-listed infections [8].

Considering the epidemiological differences in resistance mechanisms, there is a need to generate additional real-world evidence from India to examine the treatment characteristics, safety, and effectiveness of ceftazidime-avibactam against Gram-negative organisms especially MDR pathogens including carbapenem-resistant *Enterobacterales *(CRE). The objective of the current retrospective study was to understand the real-world usage, effectiveness, and safety of ceftazidime-avibactam in treating infections by MDR Gram-negative organisms.

## Materials and methods

Study design

This was a non-interventional retrospective study. Medical records of eligible hospitalized patients who had received ceftazidime-avibactam for at least 48 hours (or three doses) were considered for the study. Data was collected for the period between 1st June 2019 and 1st April 2020. The patient had to complete treatment with ceftazidime-avibactam before 1st April 2020.

Study subjects

The study focused on adult patients (≥18 years) with confirmed Gram-negative infection who received ceftazidime-avibactam as a part of their standard clinical care. Patients who had enrolled in any clinical trial/interventional study or were a part of the names access program were excluded. Patients who had received ceftazidime-avibactam for less than 48 hours and patients with documented Acinetobacter baumannii infection were also excluded.

Data was abstracted from electronic health records and individual patient medical records, wherever necessary from participating eight sites across India. Data from the patients who had received ceftazidime-avibactam as a part of the routine clinical management for Gram-negative infections was recorded as per the defined outcomes. The data was extracted by a CRO (clinical research organization), the principal investigator (PI), or a reviewer (clinical research associate) nominated by the PI. For the records missing any of the requested information, data was reported as missing. Data from patients who were part of the named access program was excluded.

Endpoints

The main objective of this non-interventional (retrospective) study was to describe the general treatment patterns, effectiveness, and safety of ceftazidime-avibactam in real-world settings.

The primary endpoints of the study were to describe the clinical and microbiological outcomes among patients treated with ceftazidime-avibactam at Day 7, Day 14/end-of-treatment (EOT) after ceftazidime-avibactam initiation, whichever was earlier. Additionally, the number of patients with serious and non-serious adverse events (AEs) associated with ceftazidime-avibactam for up to 30 days post-treatment completion with, death or discharge; whatever was first, was also documented.

The secondary endpoints included documentation of the source of infection, indication for use of ceftazidime-avibactam, dose (dosage frequency, duration, concomitant antimicrobials), causative Gram-negative organisms and their susceptibility to ceftazidime-avibactam, in-hospital length of stay (LOS) in days and incidence of recurrent infections during the hospital stay, including re-infection and relapse up to 30 days post-treatment completion with ceftazidime-avibactam, death, or discharge; whatever was first. Additionally, clinical symptom improvement or clinical symptom worsening was evaluated for patients who received ceftazidime-avibactam within 72 hours of hospital admission.

Sample size

Patients who had received at least 48 hours (complete) of ceftazidime-avibactam were screened for inclusion in the study. The data collection was conducted between 1st June 2019 and 1st April 2020. The data of all the patients recruited during this time period was abstracted.

Data analysis

The patients whose data was subjected to analysis had received ceftazidime-avibactam for at least 48 hours (or three doses). The collected data was subjected to descriptive statistical analysis.

## Results

Eight centers from various parts of the country participated in the study. A total of 189 patients were enrolled between 1st June 2019 and 1st April 2020.

The majority of the enrolled patients were males (65.1%) and had hospital-acquired infections (38.1%). Patients were diagnosed with UTIs (29.1%), IAIs (15.9%), and nosocomial pneumonia (NP) (29.1%). Nearly half of the patients (47.6%) underwent consultations with infectious disease specialists. The majority of patients were directly admitted to the ICU, with a high proportion (85.7%) requiring intensive care unit (ICU) admission. The mean Acute Physiology and Chronic Health Evaluation (APACHE) score in the overall population was 23.2 (± 16.2). Hypertension (53.0%) was the most commonly reported comorbidity, followed by diabetes (30.5%), moderate or severe renal disease (22.0%), diabetes-associated complications (18.9%), congestive heart failure (16.5%), and chronic obstructive pulmonary disease (15.2%).

The detailed patient characteristics are mentioned in Table 1.

**Table 1:** Patient characteristics at baseline (n = 189) APACHE: Acute Physiology, and Chronic Health Evaluation; DCCI: Deyo-Charlson Comorbidity Index; IAI: intra-abdominal infection; N: number of subjects in ceftazidime-avibactam group; n: number of subjects in the specified category; NP: nosocomial pneumonia; SD: standard deviation; UTI: urinary tract infection ^#^Although renal dysfunction was identified in 71 patients, evaluable data was available for 66 patients

Characteristic (Unit)	n (%)
Gender	
Male	123 (65.1)
Female	66 (34.9)
Age (Years)	Mean - 55.6 (SD: 17.8)
	Median - 57.0 (18 – 98)
Diagnosis	
UTI	55 (29.1)
IAI	30 (15.9)
NP	55 (29.1)
BSI/Sepsis	17 (34.7)
SSI	04 (08.2)
Meningitis	02 (04.1)
CLABSI	01 (02.0)
Other infections	25 (51.0)
DCCI Score (N = 164)	Mean - 3.3 (SD: 02.4)
	Median - 3.0 (0 – 11)
Source of Infection	
Hospital-acquired infection (HAI)	72 (38.1)
Healthcare-associated infection (HCAI)	65 (34.4)
Community-acquired infection (CAI)	52 (27.5)
Source of Admission	
Outpatient	42 (22.2)
Long-term care facility	19 (10.0)
Transfer from acute care hospital	23 (12.2)
Direct admission	105 (55.6)
Current Admission to ICU	
Yes	162 (85.7)
No	27 (14.3)
Renal Dysfunction	
Yes	71 (37.6)
No	118 (62.4)
Renal dysfunction severity (based on creatinine clearance)^#^	N = 66
Mild	10 (15.1)
Moderate	28 (42.4)
Severe	28 (42.4)
Indwelling devices	157 (83.1)
Urinary catheter	120 (76.4)
Intravenous peripheral catheter	77 (49.0)
Tracheal intubation	72 (45.9)
Intravenous central catheter	57 (36.3)
Arterial catheter	49 (31.2)
Arteriovenous cannula	46 (29.3)
Tracheal cannula	27 (17.2)
Any drain	16 (10.2)
Others	05 (03.2)
APACHE II Score	
Yes	85
Score	Mean – 23.2 (S. D.: 16.2)
	Median – 18.0 (0 – 72)

The majority of the patients (66.7%) received at least one antimicrobial prior to receiving treatment with ceftazidime-avibactam and 82.5% of patients used other antimicrobials concurrently. Table 2 lists the common previously used and concurrently used antimicrobials in enrolled patients.

**Table 2:** Concomitant and prior antibiotic therapy *Teicoplanin, Minocycline, Vancomycin, Antifungal, Linezolid, Chloramphenicol, Azithromycin, Ciprofloxacin, Clarithromycin, Doxycycline, Amikacin, Ceftriaxone, Clindamycin, Daptomycin, Gentamicin, Imipenem, Levofloxacin, Piperacillin-tazobactam, Rifampicin, Trimethoprim-sulfamethoxazole. ^#^Teicoplanin, Piperacillin-tazobactam, Metronidazole, Antifungal, Ceftriaxone, Amikacin, Cefoperazone-Sulbactam, Clarithromycin, Clindamycin, Doxycycline, Imipenem, Minocycline, Piperacillin, Vancomycin, Ceftaroline, Amoxicillin-clavulanate, Azithromycin, Cefuroxime, Aztreonam, Cefoperazone, Cefpodoxime, Linezolid, Moxifloxacin, Cefepime, Ciprofloxacin, Daptomycin, Dicloxacillin, Ertapenem, Flucloxacillin, Gentamicin, Levofloxacin, Rifampicin.

Generic Drug Name	n (%)
Any antimicrobials(s) used concurrently with ceftazidime-avibactam	156 (82.5)
Aztreonam	66 (42.3)
Colistin	33 (21.2)
Fosfomycin	30 (19.2)
Meropenem	22 (14.1)
Tigecycline	18 (11.5)
Metronidazole	16 (10.3)
Other*	43 (27.6)
Number of subjects with at least one prior antimicrobials	126 (66.7)
Meropenem	80 (63.4)
Colistin	39 (30.9)
Fosfomycin	26 (20.6)
Tigecycline	24 (19.0)
Other^#^	47 (37.3)

The median dose administered to the patients was 2.5g (1-2.5) given three times a day, with 6.92 days (2-19) as the mean duration of therapy. Patients with renal dysfunction (n = 66) received the ceftazidime-avibactam dose based on the degree of dysfunction. The most common dose given to patients with renal dysfunction was 2500 mg (46/66) three times a day, followed by 1250 mg (13/66), 2000 mg (4/66), and 2250 (1/66).

The mean hospital LOS was 23.1 days, and the mean ICU LOS was 15.7 days. Further details are mentioned in Table 3.

**Table 3:** Length of hospital stay; ICU stay and duration of therapy LOS: length of stay; SD: standard deviation

Duration of Administration (hours; n = 189)	Mean - 2.1 (SD: 0.8)
	Median – 2.0 (0.5-3)
Total Duration of therapy (days; n = 179)	Mean – 6.92 (SD: 4.1)
	Median – 6.0 (2-19)
Hospital LOS (days; n = 180)	Mean – 23.1 (SD: 15.1)
	Median – 20 (2-70)
ICU LOS (days; n = 155)	Mean – 15.7 (SD: 14.3)
	Median – 11 (1-69)

Among the 189 patients, 96 pathogens were identified from samples of 84 patients before initial antibiotic therapy. *K. pneumoniae* was the most commonly isolated organism in 60.7% (n = 57) followed by *E. coli* in 15.9% (n = 15) isolates. Resistant genotypes were identified in 16 samples using molecular genotyping. The most common genotypes isolated were SHV, CTX-M, and OXA-48-like in 25% of samples. Table 4 summarizes the further details.

**Table 4:** Isolated pathogens and identified resistance mechanisms *Before initial antibiotic therapy (primary infection)

Pathogens Identified	n (%)
Pathogens isolated*	94
*Klebsiella pneumoniae*	57 (60.7)
*Escherichia coli*	15 (15.9)
*Pseudomonas aeruginosa*	11 (11.7)
*Klebsiella spp.*	04 (4.2)
*Enterobacter spp.*	01 (1.0)
*Enterobacter cloacae*	01 (1.0)
*Proteus mirabilis*	01 (1.0)
*Pseudomonas spp.*	01 (1.0)
Others, bacterial agents	03 (3.1)
Genotyping done	16
SHV, CTX-M, OXA-48-like	04 (25)
CTX-M, NDM, VIM	02 (12.5)
CTX-M, NDM	02 (12.5)
NDM	02 (12.5)
CTX-M, NDM, and OXA-48-like	01 (6.2)
SHV, CTX-M	01 (6.2)
SHV, CTX-M, NDM	01 (6.2)
CTX-M, OXA-48-like	01 (6.2)
Others	02 (12.5)

Clinical outcomes were evaluated in all 189 subjects. Outcomes were recorded at 72 hours in 103 patients, on Day 7 in 83 patients, and on Day 14 or EOT in 59 patients. Recurrence of infection was observed in 4.2% of patients (8/189) and an in-hospital mortality rate of 29.6% (56/189) was observed among all patients.

The clinical outcomes are presented in Table 5.

**Table 5:** Clinical outcomes EOT: end of treatment *Patients received ceftazidime-avibactam treatment within 72 hours of hospital admission

	Time Points of Evaluation	Outcomes’ Assessment	n/N (%)
All subjects	72 Hours (Day 3)	Assessed	103/189 (54.5)
	Clinical symptom assessment	Improved	82/103 (79.6)
		Worsened	21/103 (20.4)
	Day 7	Assessed	83/189 (43.9)
	Outcomes	Clinical success	66/83 (79.5)
		Clinical failure	12/83 (14.5)
		Clinical indeterminate	05/83 (06.0)
	Day 14/EOT	Assessed	59/189 (31.2)
	Outcomes	Clinical success	45/59 (76.3)
		Clinical failure	13/59 (22.0)
		Clinical indeterminate	01/59 (01.7)
All subjects (Exposure within 72 hours of admission)	72 Hours (Day 3)*	Assessed	32/57 (56.1)
	Clinical symptom assessment	Improved	29/32 (90.6)
		Worsened	03/32 (9.4)
	Day 7	Assessed	21/57 (36.8)
	Outcomes	Clinical success	17/21 (81.0)
		Clinical failure	3/21 (14.3)
		Clinical indeterminate	01/21 (4.7)
	Day 14/EOT	Assessed	20/57 (35.1)
	Outcomes	Clinical success	19/20 (95.0)
		Clinical failure	01/20 (5.0)
		Clinical indeterminate	0/20 (0.0)
Monotherapy patients (n = 33)	72 Hours (Day 3)*	Assessed	19/33 (57.6)
	Clinical symptom assessment	Improved	16/19 (84.2)
		Worsened	03/19 (15.8)
	Day 7	Assessed	21/33 (63.6)
	Outcomes	Clinical success	18/21 (85.7)
		Clinical failure	02/21 (9.5)
		Clinical indeterminate	01/21 (4.8)
	Day 14/EOT	Assessed	09/33 (27.3)
	Outcomes	Clinical success	07/09 (77.8)
		Clinical failure	02/09 (22.2)

The samples collected from patients were subjected to microbiological evaluation to assess microbiological success or failure. Among 189 patients, 50 were assessed on Day 7, and 68 were assessed on Day 14 or EOT. Microbiological success was observed in 76.0% of patients on Day 7 and 60.3% of patients on Day 14/EOT. Please refer to Table 6 for further details.

**Table 6:** Microbiological outcomes EOT: end of treatment

Time Point of Evaluation	Microbiological Evaluation	n/N (%)
Day 7	Microbiological evaluation available	50/189 (26.5)
	Unevaluable	72/189 (38.1)
	Unknown status	67/189 (35.4)
Microbiological evaluation	Microbiological success	38/50 (76.0)
	Microbiological failure	05/50 (10.0)
	Emergent infections	07/50 (14.0)
Day 14/EOT	Microbiological evaluation available	68/189 (36.0)
	Unevaluable	51/189 (27.0)
	Unknown status	70/189 (37.0)
Microbiological evaluation	Microbiological success	41/68 (60.3)
	Microbiological failure	19/68 (27.9)
	Emergent infections	08/68 (11.8)
Monotherapy patients (n = 33)		
Day 7	Microbiological evaluation available	07/33 (21.2)
	Unevaluable/Unknown	26/22 (78.8)
Microbiological evaluation	Microbiological success	07/07 (100.0)
	Microbiological failure	0 (0)
	Emergent infections	0 (0)
Day 14/EOT	Microbiological evaluation available	06/33 (18.2)
	Unevaluable/Unknown	27/33 (81.8)
Microbiological evaluation	Microbiological success	04/06 (66.7)
	Microbiological failure	02/06 (33.3)
	Emergent infections	0 (0)

Susceptibility to ceftazidime-avibactam was tested in 43 samples, out of which 30 were found to be susceptible. E-test was the most commonly used method for 37/40 samples and yielded a minimum inhibitory concentration(MIC) of 2 mg/L; range - 0.016 to 256. The results have been summarized in Table 7.

**Table 7:** Susceptibility to ceftazidime-avibactam MDR: multi-drug resistant; MIC: minimum inhibitory concentration MIC breakpoints as per the European Committee on Antimicrobial Susceptibility Testing (EUCAST v14.0) guidelines for ceftazidime-avibactam are: for susceptibility - ≤8 mg/L and for resistance - >8 mg/L for both *Enterobacterales *and *Pseudomonas* ​​​​​​*aeruginosa*. *Susceptibility testing was conducted for 43 patients; however, evaluable data was available for 40 patients, and data for three patients was invaluable and hence excluded.

Susceptibility	Ceftazidime-avibactam (N = 189)
Done	43 (22.8%)
Susceptible	40*
Yes	30 (75.0%)
No	10 (25.0%)
Method Used	
Disc	03 (7.5%)
E-Test	37 (92.5%)
If E-test, MIC value	37
	Median - 2.0 (0.016 - 256)
Susceptible organisms	n (%) distribution
Klebsiella pneumoniae	20 (66.7%)
Pseudomonas aeruginosa	09 (30.0%)
Enterobacter aerogenes	01 (3.3%)

No AEs (including serious AEs) were reported with the use of ceftazidime-avibactam in medical records during analysis.

## Discussion

The resistance to Gram-negative pathogens is on the rise and has reached a crucial point, with pathogens exhibiting resistance to most of the available antibiotics in addition to the drying up antibiotic pipeline. The combination of ceftazidime and avibactam provides a therapeutic option for the treatment of MDR Gram-negative bacteria. Avibactam covers a wide spectrum of β-lactamases class A (ESBL, TEM, CTX-M, and KPC), class C (AmpC), and certain class D β-lactamases such as OXA-48 [7, 9]. The combination is effective against organisms that are resistant to cephalosporins and carbapenems due to the production of ESBL, AmpC cephalosporinases, and serine carbapenemases [7].

In this real-world study, 189 patients participated from eight centers between 1st June 2019 and 1st April 2020. The mean age of the population was 55.6 years and most of them were males (65.1%). Among the included patients, 48.5% were admitted to the ICU [6]. A non-interventional medical chart review study conducted in 11 countries across Europe and Latin America (LATAM) (termed EZTEAM), described patterns of use and effectiveness of ceftazidime-avibactam in real-world clinical practice. The majority of the patients in EZTEAM were male (68.2%) and the median age of the enrolled population was 62 years [10].

In the present study, UTIs (29.1%) and NP (29.1%) were the most commonly diagnosed etiologies followed by IAIs (15.9%). Rathish et al. reported bacteremia as the most commonly diagnosed infection in 48% of patients, followed by pneumonia and IAI (10% each) [6]. Similar to the present study, EZTEAM reported NP to be the most common etiology in 22.1% of patients [10]. In India, the incidence of NP in ICU ranges from 9% to 60% and is associated with a high mortality rate [11,12]. The most commonly isolated pathogen in NP is *K. pneumoniae*.

Ceftazidime-avibactam is approved for use in adults in India, for the treatment of cIAI, cUTI, and NP (including VAP) caused by Gram-negative bacteria, and bacteremia secondary to these infections [8,13]. The efficacy and safety of ceftazidime-avibactam have been demonstrated in several clinical studies [14,15] and real-world evidence studies [6,16]. In the subset analysis of the RECLAIM (Efficacy and Safety of ceftazidime-avibactam Plus Metronidazole Versus Meropenem in the Treatment of Complicated Intra-abdominal Infection) and the REPROVE (Ceftazidime-avibactam versus meropenem in NP, including VAP) studies, the efficacy and safety results of our study were found to be concurrent with the observations reported in the respective global studies [14,15]. Hereby these analyses established the effectiveness of ceftazidime-avibactam in Indian patients.

The median dose administered to the patients was 2.5 g (1 - 2.5) thrice daily with 6.92 days as the mean duration of therapy, whereas Rathish et al. reported the mean treatment duration to be 8.1 days [6]. In the EZTEAM study, the median daily dose of ceftazidime-avibactam was 6.0 g with a median duration of treatment of 9 days [10]. The mean treatment duration in our study was observed to be similar to that of previously conducted studies.

Among the 189 patients, on Day 3, improvement of clinical symptoms was recorded in 79.6% (82/103) patients. On Day 7, clinical success was achieved in 79.5% (66/83) of assessed patients. In the EZTEAM study, treatment success was achieved in 77.3% of patients [10]. Jorgensen et al. reported clinical failure in 29.1% of patients (clinical success in 70.9% of patients) treated with ceftazidime-avibactam [17]. The CAVICOR study reported an overall clinical response rate of 84.9% by the end of the study [18]. Rathish et al. reported clinical cures in 73% of CRE patients treated with ceftazidime-avibactam in a similar age group patient population [6]. It is thus evident that the effectiveness observed in our study is similar to the one reported in global and other Indian studies.

In the present study, out of 189 enrolled patients, 57 (30.16%) patients received ceftazidime-avibactam within 72 hours of hospital admission. On Day 3, 90.6% of patients who received ceftazidime-avibactam within 72 hours, showed improvement in the symptoms. On Day 7, clinical success was noted in 81%, and on Day 14/EOT, clinical success was reported in 95% of patients. It should be noted that the cohort of patients who received ceftazidime-avibactam within 72 hours was small and thus, statistical significance was not calculated. In the real-world experience with ceftazidime-avibactam, reported by Jorgensen et al., 2019, 42.9% (87/203) of patients received ceftazidime-avibactam treatment within 72 hours of admission. The clinical success rate for these patients was reported to be 44.4%, and the failure rate was 39.0% [17]. Early use of ceftazidime-avibactam (within 48 hours of infection onset) was associated with improved clinical outcomes [17]. Similarly, our study indicates that the use of ceftazidime-avibactam within 72 hours of admission has shown good outcomes in the appropriate set of patients, although statistical significance was not assessed. Furthermore, in the present study, a total of 33 patients received ceftazidime-avibactam as monotherapy. Among these patients, on Day 3, improvement in clinical symptoms was observed in 84.2% (16/33) patients. On Day 7, clinical success was reported in 18 (85.7%) patients while on Day 14/EOT, clinical success was reported in 7 (77.8%) patients. The EZTEAM study reported clinical success in 81% of patients receiving monotherapy [10]. Other studies have reported a lower mortality rate with ceftazidime-avibactam monotherapy [18, 19].

In this study, microbiological success was reported in 76% (38/50) of patients on Day 7 and in 60.3% (41/68) of patients on Day 14 or EOT. In the CAVICOR study, a microbiological eradication rate of 83.3% was reported with the use of ceftazidime-avibactam [18]. Although the number of patients evaluated for microbiological outcomes was fewer compared to other studies, the results were found to be comparable.

Among the 189 enrolled patients, 71 patients (37.6%) were reported to have renal dysfunction. Of these, only 38/66 (57.6%) of patients received the adjusted dose as per the degree of dysfunction. A similar trend was noted in another Indian study in which renal dose adjustment was done for 64.9% of patients [16]. The recommended dosage regimen for ceftazidime-avibactam is 2500 mg every 8 hours for patients with CrCl >50 ml/min across all approved indications and the dose is modified for patients with CrCl ≤50 ml/min as per prescribing information [20]. In the present study, dose adjustment was considered for 66 patients with renal dysfunction, among which 69.7% (46/66) received 2500 mg thrice daily ceftazidime-avibactam. Considering, that ceftazidime-avibactam is excreted renally, dosage modification is required in patients with renal dysfunction.

Among the 189 patients, pathogens were identified from samples of 84 patients. *K. pneumoniae* was the most commonly isolated organism in 60.6% of samples followed by *E. coli *in 15.9% of samples. In Rathish et al., 2021, 48% (n = 49/103) of the total positive cultures determined the causative organism to be *K. pneumoniae* followed by *E. coli *in 6% (n = 6/103) of cultures and *P. aeruginosa* in 4% (n = 4/103) of cultures [6]. Among the isolated *Klebsiella spp *pathogens, 32.1% of isolates were found to be susceptible to meropenem. Similarly, among the isolated *E. coli* pathogens, 55.6% of isolates were susceptible to meropenem. Among the total isolates tested for susceptibility, 75% were found to be susceptible to ceftazidime-avibactam. EZTEAM reported 7.8% of isolates from the study to be resistant to ceftazidime-avibactam [10]. Additionally, previous reports from India report the susceptibility rate of *K. pneumoniae* against meropenem to be 45% in all specimens except urine and feces. Similarly, the susceptibility rate of *E. coli* was reported to be 69% against meropenem [2].

During the study period, there was limited awareness with regards to molecular typing in India, and was not practiced in all institutes. In the present study, the most common genotypes isolated from the identified organisms were SHV, CTX-M, and OXA-48-like. The tested isolates showed the presence of SHV, CTX-M, OXA-48-like, and NDM genotypes. NDM was observed to be present along with CTX, CTX-M, VIM, and OXA-48 like while OXA-48-like was detected alongside SHV and CTX-M in addition to NDM. However, a previous Indian study had reported the presence of OXA 48 (33.61%), NDM+OXA 48 (37.81%), and NDM (28.57%) in the tested isolates [16]. The Indian Council of Medical Research (ICMR) report mentions that among *K. pneumoniae* isolates, the most commonly detected resistance mechanism was SHV (72%), followed by CTXM-15 (53%), TEM (46%), NDM (40%), and OXA-48 (39%). Similarly for *E. coli*, the most commonly detected resistance mechanism was CTXM-15 (47%), followed by TEM (37%), IMP (37%), and CIT (36%) [2]. Considering the current role of rapid diagnostics, the identification of CREs is crucial for targeted therapies like ceftazidime-avibactam. Due to the limited samples used for genotyping in this study, it is important to interpret the results cautiously and clinicians should consider local epidemiology and genotyping data in their decision making.

The mean hospital LOS was 23.1 days, and the mean ICU LOS was 15.7 days. EZTEAM study reported a mean LOS of 30.9 days from the start of ceftazidime-avibactam [10]. Other studies have reported a huge variability in hospital stays ranging from 22 to 151 days [21-25].

In our study, the overall in-hospital mortality during the study period was 29.6% which was comparable to 27% of CRE patients treated with ceftazidime-avibactam as reported by Rathish et al. [6]. In the study by Jorgensen et al., an in-hospital mortality of 16.7% was observed in patients treated with ceftazidime-avibactam [17]. The overall mortality in other global studies ranged from 8% to 23.1% [10,17-19]. Increased mortality may be associated with the higher severity indicated by an elevated APACHE II score. A difference in patient characteristics of the enrolled patients may also contribute to the mortality rate.

In the present study, no serious or non-serious AEs were reported with ceftazidime-avibactam in medical records during analysis.

The present study has a few limitations. Although this was a multicentric study representing a diverse patient pool, the retrospective and observational nature of the study limits the extent of the data that can be retrieved. Observational studies are dependent on complete and accurate patient records for data extraction and analysis. The study however adds to the existing knowledge base on the use of ceftazidime-avibactam in India.

## Conclusions

In conclusion, this study provides real-world evidence on treatment patterns and clinical outcomes associated with ceftazidime-avibactam in India. The study also complements the previously reported literature on the real-world use of ceftazidime-avibactam. The study results suggest that ceftazidime-avibactam is an effective and tolerable option for the management of Gram-negative infections in critically ill patients.
